# New Generation Sensor Web Enablement

**DOI:** 10.3390/s110302652

**Published:** 2011-03-01

**Authors:** Arne Bröring, Johannes Echterhoff, Simon Jirka, Ingo Simonis, Thomas Everding, Christoph Stasch, Steve Liang, Rob Lemmens

**Affiliations:** 1 Institute for Geoinformatics, University of Muenster, Weseler Strasse 253, 48151 Muenster, Germany; E-Mails: thomas.everding@wwu.de (T.E.); christoph.stasch@wwu.de (C.S.); 2 Faculty ITC, University of Twente, Hengelosestraat 99, 7514 AE Enschede, The Netherlands; E-Mail: lemmens@itc.nl (R.L.); 3 52° North Initiative for Geospatial Open Source Software, Martin-Luther-King-Weg 24, 48155 Muenster, Germany; E-Mail: jirka@52north.org (S.J.); 4 International Geospatial Services Institute, Werner-Heisenberg-Str. 73, 26723 Emden, Germany; E-Mails: johannes.echterhoff@igsi.eu (J.E.); ingo.simonis@igsi.eu (I.S.); 5 Department of Geomatics Engineering, University of Calgary, Calgary, AB, T2N 1N4, Canada; E-Mail: steve.liang@ucalgary.ca (S.L.)

**Keywords:** Sensor Web Enablement, SWE, OGC, sensor observation service, sensor planning service, observations & measurements, geosensor networks

## Abstract

Many sensor networks have been deployed to monitor Earth’s environment, and more will follow in the future. Environmental sensors have improved continuously by becoming smaller, cheaper, and more intelligent. Due to the large number of sensor manufacturers and differing accompanying protocols, integrating diverse sensors into observation systems is not straightforward. A coherent infrastructure is needed to treat sensors in an interoperable, platform-independent and uniform way. The concept of the Sensor Web reflects such a kind of infrastructure for sharing, finding, and accessing sensors and their data across different applications. It hides the heterogeneous sensor hardware and communication protocols from the applications built on top of it. The Sensor Web Enablement initiative of the Open Geospatial Consortium standardizes web service interfaces and data encodings which can be used as building blocks for a Sensor Web. This article illustrates and analyzes the recent developments of the new generation of the Sensor Web Enablement specification framework. Further, we relate the Sensor Web to other emerging concepts such as the Web of Things and point out challenges and resulting future work topics for research on Sensor Web Enablement.

## 1. Introduction: From Heterogeneous Sensors to the Sensor Web

A *sensor* is defined from an engineering point of view as a device that converts a physical, chemical, or biological parameter into an electrical signal [1]. Common examples include sensors for measuring temperature (*i.e*., a thermometer), wind speed (an anemometer) conductivity, or solar radiation. While a sensor is the most basic unit, a *sensor system* is an aggregation of sensors, attached to a single platform [2]. Examples are a weather station with attached sensors, or a combination of heart frequency and blood pressure sensors carried by a human or animal. A sensor or a sensor system may be abstracted as a *sensor resource*. A *sensor network* consists of a number of spatially distributed and communicating sensor resources [3].

Sensor technology is continuously improving as the devices become smaller, cheaper, more intelligent, and more power efficient. In consequence, more and more application fields are making use of these technologies. Examples are disaster management, environmental monitoring, precision agriculture, early warning systems, home as well as public security, or human health [4–6]. The kinds of sensor resources utilized in these applications may be stationary or in motion and could gather data in an *in-situ* or remote manner. Due to the large variety of sensor protocols and sensor interfaces, most applications are still integrating sensor resources through proprietary mechanisms, instead of building upon a well-defined and established integration layer. This manual bridging between sensor resources and applications leads to extensive adaption effort, and is a key cost factor in large-scale deployment scenarios [7].

This issue has been the driving force for the Open Geospatial Consortium (OGC) to start the Sensor Web Enablement (SWE) initiative (*http://www.ogcnetwork.net/swe*) back in 2003. Within the SWE working group a suite of standards has been developed which can be used as building blocks for a *Sensor Web.* SWE defines the term Sensor Web as “Web accessible sensor networks and archived sensor data that can be discovered and accessed using standard protocols and application programming interfaces” [8]. First described by Delin *et al.* in 1999 [9], a *Sensor Web* was considered as an autonomously organized wireless sensor network which can be deployed to monitor environments. As a smart macro instrument for coordinated sensing [10], Delin’s Sensor Web concept consists of sensor nodes which not only collect data, but also share their data and adjust their behaviour based on that data. Thereby, the term “Web” within Delin’s “Sensor Web” relates to the intelligent coordination of the network rather than the World Wide Web (WWW) [11]. Later, the meaning of “Sensor Web” changed and it was more and more seen as an additional layer integrating sensor networks with the WWW and applications [12–14]. Today, the notion of “Sensor Web” has been largely influenced by the developments of the SWE initiative. It is defined as an infrastructure which enables an interoperable usage of sensor resources by enabling their *discovery*, *access*, *tasking*, as well as *eventing* and *alerting* within the Sensor Web in a standardized way. Thus, the Sensor Web is to sensor resources what the WWW is to general information sources—an infrastructure allowing users to easily share their sensor resources in a well-defined way [15]. It hides the underlying layers, the network communication details, and heterogeneous sensor hardware, from the applications built on top of it.

To achieve this, SWE incorporates models for describing sensor resources and sensor observations. Further, it defines web service interfaces leveraging the models and encodings to allow accessing sensor data, tasking of sensors, and alerting based on gathered sensor observations. The SWE specifications provide the functionality to integrate sensors into Spatial Data Infrastructures (SDI). The integration of sensor assets into SDIs makes it possible to couple available sensor data with other spatio-temporal resources (e.g., maps, raster as well as vector data) at the application level, which maximizes the information effectiveness for decision support. Due to this integration, Sensor Webs and the geosensors they comprise represent a real-time link of Geoinformation Systems (GIS) into the physical world. Thereby, *geo*sensors are defined as sensors delivering an observation with georeferenced location [2].

This work builds upon but is different from [3,8,16]. While those former papers in this research area describe the architecture of the first generation of OGC’s SWE specification framework, this article goes beyond that and analyzes the *new generation SWE* by pointing out differences with the preceding versions of the standards and by describing newly introduced specifications (Section 3). Before we describe those changes, this work relates SWE to other approaches for linking sensor resources to the Web (Section 2). Section 4 sketches how the SWE services can be applied to build a Sensor Web infrastructure and presents conducted projects which utilized the SWE framework. In Section 5, we identify challenges and future work for SWE including the improvement of interoperability, the integration of sensors and services, and new paradigms such as humans as sensors and the Semantic Sensor Web. The article ends with a conclusion in Section 6.

## 2. Related Work on Bridging Between Sensors and Applications

Goal of the Sensor Web research field is to bring sensor resources on the Web and make them available to applications. To achieve this middleware technologies, which help to manage the heterogeneity of sensor resources and make them usable on the application level, have been developed. This section gives an overview of the broader research area, comes up with a categorization of different middleware classes, lists selected approaches, and points out their characteristics. Some of the listed approaches use the Sensor Web Enablement standards, other solutions incorporate non-standardized interfaces. The categorization below provides an overview and helps readers to find solutions that fit the needs of their use cases.

The Sensor Web can be considered as a middleware between sensors and applications. This implies three main architectural layers. First, there is the *sensor layer*, where the actual hardware devices reside and various kinds of proprietary or standardized communication protocols are used by different sensor types (e.g., WPAN protocols, IEEE 1451). Second, there is an intermediary *Sensor Web layer* providing functionality to bridge between sensor resources and applications. On top, there is the *application layer* where direct interaction with clients (human end users or computers) takes place. Applications could run on various client devices ranging from cell phones to server machines. The three main layers are further divided into sub-layers depending on the architectural design of middleware systems.

**Figure 1.**
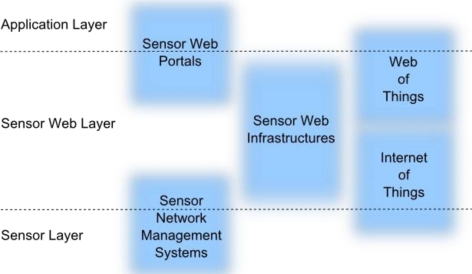
The Sensor Web layer stack and located middleware classes.

Figure 1 shows the described layer stack and places four identified middleware classes on their positions within the layer stack. Note that the borders of those middleware classes are drawn fuzzy since their functionalities might overlap and some middleware approaches offer functionalities belonging to multiple classes. Also, middleware solutions can be built upon each other to realize the entire Sensor Web layer stack. The four identified middleware classes are described in the following.

### 2.1. Middleware for Sensor Network Management Systems

Research on integrating sensors with applications begins on the lowest level, namely with research on middleware concepts which manage the communication within sensor networks. Due to their advanced functionality and the resulting challenges, *wireless* sensor networks (WSNs) are of particular interest. Foundational work on managing WSNs includes research areas such as routing protocols [17,18], optimization of in-network communication [19], coverage optimization of sensor networks [20,21], the optimization of data collection paths [22], and the localization of sensors within a network [23,24].

Such basic functionality for managing sensor networks is provided by WSN middleware. A comprehensive survey on WSN middleware approaches is given by Wang *et al.* [25]. Examples for WSN middleware solutions are MundoCore [26], Mires [27] or MiLAN [28]. Such middleware approaches which serve sensor network management functionality do not focus on enabling easy access to sensors for applications. Hence, they can be considered as closer to the lower sensor layer and do not fully reach out to the application layer as depicted in Figure 1. However, such middleware solutions may serve as the basis for other approaches such as Sensor Web infrastructures.

### 2.2. Middleware for Sensor Web Infrastructures

This class comprises middleware solutions which are particularly designed for making sensors available on the Web and enable the access to sensors from the application level by building up Sensor Web infrastructures. The comprised approaches abstract from details of the sensor network and (usually) do not provide sensor network management functionality, such as the approaches described in Section 2.1. Some of the comprised middleware approaches make use of the SWE standards to offer interoperable access to sensors. Others define their own proprietary interfaces and data encodings.

First, implementations of the SWE service specifications itself can be seen as part of this class. The 52°North Sensor Web framework (*http://52north.org/swe*) provides implementations for the different SWE services. An implementation of the Sensor Observation Service (SOS; Section 3.3.2) enables querying as well as inserting measured sensor data and metadata. While the SOS follows a pull-based communication paradigm to access sensor data, the Sensor Alert Service (SAS) and its successor the Sensor Event Service (SES; Section 3.3.4) push sensor data to subscribed clients in case of user defined filter criteria. The Sensor Planning Service (SPS; Section 3.3.3) enables tasking of sensors (e.g., setting the sampling rate of a sensor). Discovery of sensors is supported by implementations of Sensor Instance Registry (SIR) and Sensor Observable Registry (SOR; Section 3.3.5). To integrate sensor resources with the SWE service implementations, the 52°North framework comprises an intermediary layer, called the Sensor Bus [29], to which sensor resources and SWE services can be adapted to establish communication.

Other middleware systems for building Sensor Web infrastructures based on SWE are GeoSWIFT [30] and its successor GeoSWIFT 2.0 [31]. The latter redesigns the GeoSWIFT system to optimize its scalability by introducing a peer-to-peer based spatial query framework. The PULSENet framework [32], which reuses and amends the open source components of the 52°North Sensor Web framework, allows the implementation of a SWE-based Sensor Web. An important aspect of the system is to accommodate legacy and proprietary sensors (e.g., IEEE 1451 or CCSI) in SWE-based architectures. NASA’s Sensor Web 2.0 [33] system incorporates SWE services and combines them with Web 2.0 technology. It envisions an easy creation of mash-up applications which integrate data from multiple sources. This includes for example the creation of composite maps overlaying data from sensor sources with data from other sources such as weather or traffic. The mash-up functionality is realized by incorporating the representational state transfer (REST) approach [34] to access data. However, it remains unclear how the system provides REST access to sensor resources by leveraging SWE services.

Non-standardized approaches for building a Sensor Web are for example Hourglass [13], the Global Sensor Network (GSN) [7], the Sensor Network Services Platform (SNSP) [35], or SOCRADES [36]. GSN focuses on a flexible integration of sensor networks to enable fast deployment of new sensors. Its central concept is the virtual sensor abstraction with XML-based deployment descriptors in combination with data access through plain SQL queries. GSN provides distributed querying, filtering, and aggregation of sensor data as well as the dynamic adaptation of a system during runtime. Similar to GSN is Hourglass, that provides an architecture for connecting sensors to applications. It offers discovery and data processing services and focuses on maintaining the quality of service of data streams. SNSP defines a set of service interfaces usable as an application programming interface for wireless sensor networks. Similar to the SWE framework, the approach follows a top down view on sensor networks independent of a particular implementation or hardware platform. It offers (non-standardized) service interfaces for data querying and sensor tasking, but also auxiliary services for locating, timing, and a concept repository. SOCRADES comprises multiple services providing functionality such as data access, eventing or discovery. The integration of sensors into the infrastructure is done by implementing sensor gateways which hide the communication protocol and expose the sensor functionality as device level web services. In contrast to the SWE framework, the operations of individual services are not standardized. The four approaches described above do not offer service interfaces for tasking of sensors, such as the SPS. Further, only SOCRADES provides push-based delivery of sensor data, as offered by the SAS or SES.

Agent based systems for establishing Sensor Web infrastructures are for example IrisNet [12] or the Sensor Web Agent Platform (SWAP) [14]. IrisNet envisions a global Sensor Web by focusing on data collection and query answering. Therefore, it introduces *organizing agents* to store sensor data in a hierarchical, distributed database and *sensing agents* which collect the sensor data. SWAP combines the paradigms of a service oriented architecture and multi agent systems. By building on OGC’s SWE framework, the proposed architecture improves the integration of arbitrary sensors into workflows on the application level. This is done by introducing a three tier architecture comprising sensor, knowledge and application layer. Different kinds of agents residing on the three layers provide certain functionality and facilitate the development of new applications and the integration of sensors with applications.

### 2.3. Centralized Sensor Web Portals

Emerging centralized web portals for sensors can be seen as a new class of systems to enable access to sensor resources on the application level. Such Sensor Web portals enable users to upload and share sensor data. The support of data formats depends on the portal and may range from numeric data (e.g., temperature measurements) to audio and video data (e.g., from Web cameras). Uploaded data can then be queried and displayed by end users for example as time series charts or video feeds. Instances of such systems are SensorMap with its underlying SenseWeb infrastructure [37], SensorBase [38], Pachube (*http://www.pachube.com*), as well as Sensorpedia [39]. Specific subtypes of such Sensor Web portals are platforms which are specialized for certain sensor types or domains. Examples are Weather Underground (*http://www.wunderground.com*) allowing people to register their home weather station and contribute their measured data to weather forecast computations, or EarthCam (*http://www.earthcam.com*) which links the video feeds from thousands of Web cameras.

Such Sensor Web portals offer APIs to the public for registering sensors, uploading sensor data, as well as querying inserted data. Once registered, the discovery of sensors is also supported. However, a controlling or tasking of sensors, as provided by OGC’s SPS service, is usually not possible. Except for Sensorpedia, which supports the SOS as a data source [40], none of the portals leverage SWE standards.

The centralized approach of the portals is the main difference to the decentralized approaches of Sensor Web infrastructures described in Section 2.2. Metadata of registered sensors as well as uploaded sensor data are hosted by the centralized portal instead of separate service components within enterprise architectures. This may be unsuitable for use cases with needs for full control over deployment and administration set up or strict data privacy regulations.

### 2.4. Frameworks for Internet of Things/Web of Things

While the Sensor Web describes an infrastructure for heterogeneous sensors, which may be networked or individual, stationary or mobile, and can incorporate *in-situ* or remote sensing devices, the vision of the two related research fields of Internet of Things [41] and Web of Things [42] is on integrating general, real-world “things” with the Internet or Web, respectively. Examples for such things are household appliances, embedded and mobile devices, but also smart sensing devices. Often, the user interaction takes place through a cell phone acting as the mediator within the triangle of human, thing, and Internet/Web. The application fields of the Internet of Things are influenced by the idea of ubiquitous computing [43]. They reach from smart shoes posting your running performance online, over management of logistics (e.g., localization of goods in the production chain), to insurance (e.g., car insurance costs based on the actually driven kilometres).

For technically realizing the Internet of Things, research topics include protocol stacks for the Internet Protocol (IP) standard optimized for smart things (e.g., IPv6, 6LoWPAN) [44], naming services for things [45], or the unique identification of objects (e.g., RFID). The Web of Things can be seen as an evolvement of the Internet of Things. It leverages existing Web protocols as a common language for real objects to interact with each other. HTTP is used as an application protocol rather than a transport protocol as it is generally the case in web service infrastructures such as OGC’s SWE framework. Things are addressed by URLs and their functionality is accessed through the well-defined HTTP operations (GET, POST, PUT, *etc.*). Hence, Web of Things applications follow the REST paradigm [46]. Specific frameworks (e.g., [47,48]) offer REST APIs to enable access to things and their properties as resources. These REST APIs may not only be used to interact with a thing via the Web, also website representations of things may be provided to display dynamically generated visualizations of data gathered by the thing. Then, the mash-up paradigm and tools from the Web 2.0 realm can be applied to easily build new applications. An example application may use Twitter to announce the status of a washing machine or may let a fridge post to an Atom feed to declare which groceries are about to run out.

However, complex use cases which need detailed and standardized sensor information models and richer functionality on access, discovery, tasking and event handling, such as disaster management or early warning systems, may not be realizable with the Web of Things approach. On the other hand, the integration of smart things into a standardized web services architecture might be too costly and complex in practical applications for simple objects [49]. Approaches such as NASA’s Sensor Web 2.0 (Section 2.2) try to combine SWE and Web 2.0 and thereby integrate aspects of the Web of Things with SWE.

## 3. New Generation Sensor Web Enablement

This section illustrates the current state of OGC’s SWE specification framework. It builds upon existing work (e.g., [3,8,16]) which has given an overview of the initial version of SWE. This work goes beyond those papers by analyzing the recent developments and pointing out the major conceptual changes which have been applied to evolve the SWE framework (SWE 1.0) to what we call here the *New Generation Sensor Web Enablement* (SWE 2.0). Also, new concepts, which are currently being discussed or considered as best practice but have not reached the standard approval yet, are described and analyzed regarding their potential relevance in future. Further, relations between the existing specifications are depicted which help the reader to apply SWE in the right way. This section intentionally does not describe the SWE specifications in technical depth; the interested reader is pointed to the original OGC specification documents.

### 3.1. Introduction to OGC’s Sensor Web Enablement Initiative

The *Open Geospatial Consortium* (OGC) is an international, non-profit standardization organization comprising over 400 companies, governmental agencies and universities. Those members are participating in a consensus process to develop standards for data models and (Web) services for enabling the *Geospatial Web* which integrates the World Wide Web with spatiotemporal data and services.

The OGC SWE working group was founded in 2003. As part of OGC's specification program, the SWE working group develops standards to integrate sensors into the Geospatial Web for enabling a specialized subtype, the *Sensor Web*. Therefore, SWE has specified a number of standards defining formats for sensor data and metadata as well as service interfaces which enable the interoperable access to real and virtual sensor resources (simulation models are examples for virtual sensor resources [50]). The SWE 1.0 specifications have been approved as standards between 2006 and 2007. They offer the following functionalities:

- *Description of sensor data* to enable further processing.
- *Description of sensor metadata* including properties and behavior of sensors, as well as correlating reliability and accuracy of collected measurements.
- *Access to observations and sensor metadata* based on standardized data formats and appropriate query and filter mechanisms.
- *Tasking of sensors* for the acquisition of measurement data.

Further, the following functionalities are supported by the first generation SWE, but are not yet approved as standards:

- *Alerting* based on sensor measurements and defined alert criteria.
- *Notification* of end users in case of alerts or finished sensor tasks via e.g., SMS or e-mail.

The new generation of SWE adds further functionalities to the SWE framework, which is also not yet approved as standards:

- *Eventing* mechanisms which advance the basic alerting functionality of the first generation SWE specifications.
- *Discovery* of sensor resources and sensor observables.

To provide the above mentioned functionalities the specifications of the first as well as the new generation SWE are divided into two informal subgroups. First, the *information model* includes the data models and encodings. Second, the *interface model* comprises the different web service interface specifications (the interface model was formerly called service model and to avoid naming confusion with the SWE Service Model standard [51], which is part of the new generation SWE, we propose this renaming). In the next subsections, the two parts of the SWE framework are described, it is illustrated how the functionalities listed above are realized, and the changes from first to new generation SWE are analyzed.

A non-functional, but formal change which concerns all new SWE standards or candidate standards is the application of OGC’s new modular specification model [52]. By applying these guidelines to structure and design the specification document, numerous *requirements* are formulated throughout the specifications. These requirements convey criteria which need to be fulfilled if compliance with the standard is claimed. This improves the strictness of a specification and facilitates conformance testing of compliant software components. However, only very few OGC standards which are compliant with this new model have been published yet, therefore, experiences regarding ease of use and level of achieved interoperability are still outstanding. Nevertheless, it is expected that the advantages of the new specification model overcompensate the disadvantages such as degraded readability and the explosion of standard production costs.

### 3.2. Evolvement of SWE Information Model

**Figure 2.**
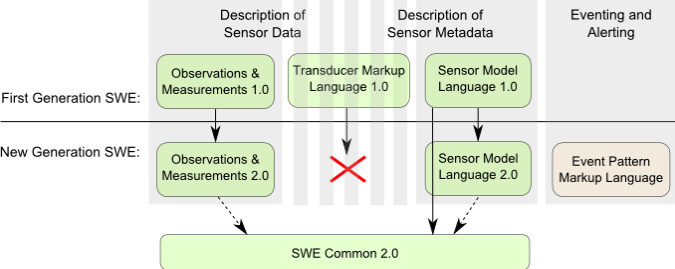
Evolvement of the SWE information model. *Green boxes*: specifications approved as standards (or in standardization process); *Beige boxes*: discussion papers; *Solid arrows*: “evolvement to”; *Dashed arrows*: “dependent on”.

The SWE information model comprises a set of standards which define data models primarily for the encoding of sensor observations as well as sensor metadata. For this purpose, the first generation of SWE contained three specifications: Observations & Measurements (O&M) [53,54], the Sensor Model Language (SensorML) [55], and the Transducer Markup Language (TML) [56]. Figure 2 shows the evolvement of the first generation SWE information model to its current state. In the new generation SWE, O&M 1.0 which is used for the description of measured sensor data evolves to O&M 2.0 as described in Section 3.2.2. Also, the SensorML 1.0 standard advances to version 2.0. Although the work on SensorML 2.0 is still in progress, we outline the expected changes in Section 3.2.3. Further, the SWE Common data model, which defines data types shared by multiple SWE specifications, is extracted from the SensorML standard and is provided as a standalone specification called SWE Common 2.0 (Section 3.2.1).

TML supports the encoding of sensor data as well as metadata by focusing on data streaming. TML has only been rarely used in practice and has not been further evolved so far. In the new generation of SWE specifications, TML is not referenced anymore and recent conversations in OGC’s SWE working group showed that there is no urgent demand in TML and a retirement of the standard is in discussion. Hence, the authors do not see TML as part of the new generation SWE.

Another new specification is the Event Pattern Markup Language (EML) [57]. It is used to define event patterns as processing rules for Complex Event Processing (CEP) and Event Stream Processing (ESP). These processing techniques can be implemented within services such as the Sensor Alert Service or the Sensor Event Service (Section 3.3.4).

Next, the advancements of the SWE information model are analyzed. Table 1 summarizes the main changes from the first to the new generation of the SWE information model. These changes are detailed in the next sections.

#### 3.2.1. Common SWE Data Types

Common and basic data types used throughout the SWE framework are defined by SWE Common. The first version of the SWE Common data model and its encoding was defined within the document of the SensorML 1.0 standard. Data types defined in this data model are used as input parameters of SWE service operations or as the basis for complex types.

In the first generation SWE, this common model was strongly used in SensorML, but also the interface models of SOS 1.0, SPS 1.0, and SAS referenced the SWE common data types. In O&M 1.0, it was also possible to use SWE Common for the encoding of observation results; however, there was no formal dependency between these two specifications. In the new generation of SWE, the position of SWE Common 2.0 [58] is strengthened. It is specified in its own standard document, independent of SensorML, and is formally referenced by the new SWE specifications.

The main goal of SWE Common 2.0 is to enable the description and provision of *data* in an interoperable way. Therefore, the data model contains four main pieces of information: *representation* of data values (e.g., categorical, numeric, or textual), *nature* of data by referencing to semantic descriptions, *quality* of data, and data *structure* defining how individual pieces of data are grouped, ordered, and repeated to form a complete data stream. Besides simple data component types (e.g., Text or Boolean), SWE Common 2.0 contains aggregate types (e.g., Record, or Vector). Instances of aggregate types can carry multiple data components, for example to describe the structure of a sensor data stream. The individual data components have properties to define their quality or link to their semantics stored for example as concepts in ontology repositories.

SWE Common 2.0 separates the conceptual model from its implementation. Based on the conceptual model different implementations may be defined; included in SWE Common 2.0 is an XML implementation of the model.

The definition of binary, textual and XML based data stream encodings has been improved. For example, the XML encoding now enables sensor data to be described and provided in simple XML format. Direct support for multiplexed encodings and standard encodings has been removed. However, additional encodings can be defined as extensions to the core SWE Common standard.

The model for describing observable phenomena based upon a dictionary structure defined by the Geography Markup Language (GML) [59] has been removed. This emphasizes the fact that SWE Common 2.0 does not depend on or favors a specific approach for modeling phenomena. A SWE Common data component simply references a concept that provides its definition—which can be an entry in a dictionary, thesaurus, or ontology.

Data types for the definition of spatiotemporal properties, such as *position*, *envelope*, *curve*, or *time grid*, have been removed from the conceptual model. The according functionality can now be achieved via a soft-typed approach that uses a specific combination of aggregate data components. In general, all SWE Common types do now include an explicit extension point that can be used to add any information that is not foreseen at the moment—without breaking existing implementations.

#### 3.2.2. Description of Measured Sensor Data

The Observations & Measurements standard defines a domain independent, conceptual model for the representation of (spatiotemporal) measurement data. It comprises an implementation of this conceptual model as an XML based GML application schema. ISO defines an *application schema* as a conceptual schema for data required by one or more applications. Thus, O&M can be seen as a conceptual schema for sensor applications based upon GML. O&M is particularly used for the creation of response documents for the *GetObservation* operation of the SOS (Section 3.3.2). However, O&M can also be used as a generic means to deal with measurements in a standardized way.

The above has been the case for the first version of O&M [53,54] and is also the case for O&M 2.0. However, conceptual model and its encoding are now more strictly divided. In fact, the major objective of the development of O&M 2.0 has been to harmonize it with existing foundational ISO models and to bring it into the ISO standardization process. This aim has been achieved, and the conceptual model of O&M 2.0 has reached the status of an ISO final draft international standard [60] while its XML implementation is integrated into the more technical standards landscape of the OGC [61].

**Figure 3.**
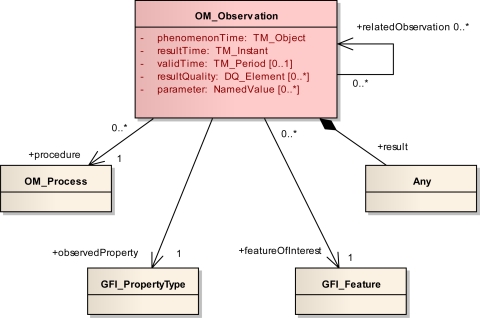
Basic observation model of O&M 2.0.

The basic observation model as designed in O&M 2.0 is shown in Figure 3. An *observation* has a relationship to a *procedure* representing the process which has performed the observation, e.g., a physical sensor or a simulation. The *observed property* points to a description of the property which is observed (e.g., “water temperature” or “salinity”). The observation’s *result* is not restricted to a certain type in the basic observation model and can be of any type, ranging from a single measurement to an n-dimensional coverage of values. Subtypes of the basic observation then restrict the type of the result. The *feature of interest*, the computational representation of a real world feature (e.g., “Gulf of Mexico” or “water gauge X at Mississippi river”) carries the property which is observed. The observation provides a value for this property at a certain time, the *phenomenon time*. The phenomenon time was formerly called sampling time and was renamed to better reflect that it represents the time when the observation’s result applies to the observed property.

In addition to the phenomenon time, an observation contains two other temporal properties: *result time* and *valid time*. The mandatory result time property represents the time when the observation’s result was produced. The valid time is an optional property introduced in O&M 2.0 that defines the time period for which the observation’s result is usable. This is for example valuable in forecasting scenarios where a weather forecast made at 9:00 may already be superseded by a new forecast made at 10:00—the valid time of the first forecast would then be the time period that starts at 9:00 and ends at 10:00.

Spatial information for an observation is usually given by a location property of the feature of interest. However, in O&M 2.0, a new spatial profile facilitates the provisioning of an observation’s sampling geometry—the spatial extent that the result of the observation applies to. This is usually the extent of the observation’s feature of interest. Without the profile, this information has to be extracted from the feature of interest, which could involve complex computations of the actual geometry and can also require dealing with previously unknown feature types.

Also newly introduced in O&M 2.0 is the *related observation* property. This property can be used to express relationships between observations. Coming back to the weather forecasting scenario, this property can be utilized to model a “supersede” relationship between two successively computed forecasting observations. Removed from the O&M data model is the data type that served as a container for collections of observations. This has been done since containers for multiple observations are now defined by the service specifications (e.g., SOS 2.0) or applications using O&M.

#### 3.2.3. Description of Sensor Metadata

For the description of sensor metadata, the SWE framework defines the Sensor Model Language (SensorML). SensorML 1.0 [55] specifies a model and XML encoding for the description of all kinds of sensor related *processes*. A process can be for example a measurement procedure conducted by a sensor or the post processing of previously gathered data. In SensorML a sensor is defined as a process which is capable of observing a phenomenon and returning an observed value. It allows a detailed description of a process including a listing of its inputs, outputs, parameters, and process methods. Further metadata of a process can be defined including its identification and classification, as well as characteristics such as the temporal availability or its spatial description. SWE services use SensorML as a format for describing their associated sensors.

Thereby, the design of SensorML 1.0 focused primarily on the following functionalities:

- Supporting the discovery of sensors by providing a means for encoding sensor metadata.
- Providing information that can be used for understanding and analyzing data produced by the sensor (e.g., the parameters of the sensor calibration).
- Allowing the description of post processing steps that were performed on sensor data so that it can be reconstructed how a data set has been created.

The work on SensorML 2.0 is currently still in progress and addition of further functionalities is planned. First, a property inheritance mechanism for SensorML shall be included. This mechanism aims at reducing the size and redundancy of sensor metadata descriptions by constructing inheritance hierarchies. Second, SensorML shall be extended to enable the precise and well-defined description of a sensor’s protocol and interface. The vision behind such a detailed description of the sensor protocol is to enable an on-the-fly integration of the sensor with the Sensor Web, by using the protocol definition to transform sensor messages to Sensor Web protocols. The description of the sensor protocol, once designed for a particular sensor type, can then be reused in different scenarios and can be shared among user communities, which facilitates the usage of SWE in general. An extension of SensorML, which allows such a declarative description of the sensor protocol, has been proposed by the Sensor Interface Descriptor (SID) concept [62,63] which may influence the development of SensorML 2.0.

Since SensorML is very generic, potential use cases cover a broad range. However, this fact makes it also necessary to define profiles for SensorML in order to ensure that every SensorML based sensor description contains all metadata for the particular use case. An example for such a profile is the SensorML profile for discovery of sensors (Section 3.3.5).

### 3.3. Evolvement of SWE Interface Model

The SWE interface model comprises standards that specify the interfaces of the different Sensor web services. Four service interfaces were defined for the first generation of SWE: The Sensor Observation Service (SOS) [64] offers pull-based access to sensor measurements as well as metadata. The Sensor Alert Service (SAS) [65] allows subscribing to alerts in case of a sensor measurement event that fulfills certain criteria. The Sensor Planning Service (SPS) [66] can be used for tasking sensors and setting their parameters. The Web Notification Service (WNS) [67] is, unlike the other three services, not directly sensor related. It is a supportive service which provides asynchronous notification mechanisms between SWE services and clients or other SWE services (e.g., delivery of notifications) including protocol transducing capabilities.

**Figure 4.**
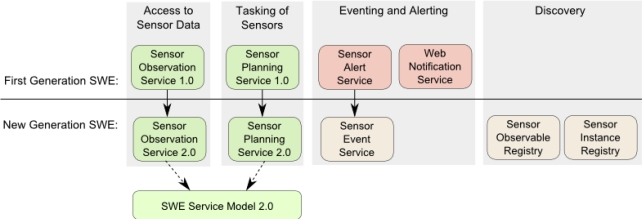
The new generation of the SWE interface model. *Green boxes*: specifications approved as standards (or in standardization process); *Red boxes*: best practice specifications which have not been approved as standard; *Beige boxes*: discussion papers; *Solid arrow*: “evolvement to”; *Dashed arrow*: “dependent on”.

In the new generation of SWE, the SOS and SPS have evolved to version 2.0, as shown in Figure 4. The conducted changes are in detail described in Sections 3.3.2 and 3.3.3. A common basis for the service development has been introduced, the SWE Service Model specification (Section 3.3.1), which serves as a foundation for SOS 2.0 as well as SPS 2.0. While the SAS has evolved to the more powerful Sensor Event Service (SES), the WNS has not yet been further developed since an approved standard for eventing needs to be in place first (Section 3.3.4). Furthermore, new service interfaces to support the sensor specific aspects of discovery have come up and are being discussed at OGC (Section 3.3.5). In the following subsections, the interface model of the new generation SWE is analyzed and its conceptual changes are highlighted.

#### 3.3.1. Common SWE Service Aspects

A major change in the design of the new generation SWE is a common model for SWE services. Many aspects of SWE service specifications can be commonly defined. This includes service operations and exceptions, among others. To harmonize these aspects the SWE Service Model (SWES) standard [51] has been developed. With the intention that SWE service specifications reference this standard, interoperability is improved through more consistent specifications and reuse of common types. So far, SPS 2.0 and SOS 2.0 are based on this common SWE service model. The main advancements introduced through this new specification are outlined in Table 2 and detailed in the following.

##### Common Capabilities Content Model

Both SPS 1.0 and SOS 1.0 used the *offering* concept to structure their service metadata. Both services had their own way of realizing the offering in their conceptual model. Despite some commonalities regarding the requirement to list the sensor identifier(s) and observed/observable properties in their offerings, SOS 1.0 and SPS 1.0 each defined their own data types to convey the information to clients. The SWE Service Model defines an *abstract offering* type that provides the information. It is reused in the conceptual models of SOS 2.0 and SPS 2.0. SWES thereby also defines a mechanism with which the amount of redundant information and therefore the size of a service’s capabilities document can be reduced significantly. This mechanism is called *property-inheritance*. It has been derived from a similar approach that OGC’s Web Mapping Service uses to reduce the size of its capabilities document.

##### Extensibility Points

The service models of the first generation of SWE lacked flexibility in a sense that they were not designed for change. The SWE Service Model adds extensibility points to relevant information entities such as operation requests and responses and other object type definitions. Developers can leverage these extensibility points by inserting their specific information items. Each of these items has to possess well-defined semantics that can influence service and/or client behavior.

##### Improved Sensor Description Management

Metadata on sensors is a very important part of SWE (Section 3.2.3). SOS 1.0 defined a *DescribeSensor* operation with which the current sensor description could be retrieved. SPS 1.0 achieved this functionality by directly referencing the description from within its capabilities document. In the new generation of SWE, this has been harmonized through a common *DescribeSensor* operation defined by SWES. This operation enables clients to retrieve not only the current description of a given sensor, but also descriptions that were valid in the past or will become valid in future. This is useful to track changes in a sensor description, regardless of the format that the description is given in—keeping in mind that SWE 2.0 does not mandate a specific description format to support the requirements of different domains, though SensorML is recommended. A common use case that is supported through such time tagged sensor descriptions is when a sensor is mobile. There, the sensor location could be provided as part of the sensor description.

Further, SWES defines the *UpdateSensorDescription* operation which allows updating sensor descriptions. Therefore, SWES defines precise rules how to handle situations in which the validity time of a submitted sensor description and an already existing sensor description temporally interact. This enables sensor providers to manage a revision history of metadata for a given sensor through a standardized interface.

##### Conceptual Models for Sensor Insertion and Deletion

Only SOS 1.0 defined an operation (*RegisterSensor*) to support the insertion of new sensors. However, the model of that operation, and in particular the association of the new sensor with offering specific metadata, was insufficient. The SWE Service Model defines the conceptual model of an *InsertSensor* operation which replaces the *RegisterSensor* operation. The *InsertSensor* operation now includes all the information items required to populate a SWE service offering (e.g., properties observable by the new sensor or features related to that sensor). Metadata specific for a certain type of SWE service (e.g., SOS or SPS) can be added via a well-defined extension point and sub typing the abstract *InsertionMetadata* parameter.

While SOS 2.0 leverages this model, neither SPS 1.0 nor SPS 2.0 enables the insertion of sensors. This is due to the fact that the implementation of an SPS, which is generic enough to support all possible realizations of tasking logic and connections to the underlying sensor system, is very difficult. The Sensor Interface Descriptor extension of SensorML aims at supporting such plug-and-play of sensors at SOS as well as SPS for certain tasking use cases [62].

Complementary to the insertion of sensors, SWES defines an operation for deleting sensors at a SWE service. While the structure of the operation is quite simple, the possible semantics attached to the operation can be fairly complex. Therefore, SWES explicitly defines the *DeleteSensor* operation as an abstract operation to let concrete service types such as the SOS 2.0 define the missing semantics.

##### Basis for SWE Service Eventing

In SWE 1.0, the SAS and SPS dealt with event handling. While SAS was designed to filter incoming sensor data, the SPS defined some events that were published to task owners while their task was performed. Neither common SWE service events were foreseen in SWE 1.0 nor a *publish/subscribe* interface common to SWE services.

The SWE Service Model fills this gap. It defines basic service events that are specific to SWE services, for example the *SensorDescriptionUpdated* event. Event channels are also defined to facilitate access and provision of SWES events. A conceptual model for notification metadata like information on the events and channels as well as filter dialects supported by a given service instance is defined by SWES. This metadata can be added to the capabilities document of a SWE service. The SPS 2.0 leverages this structure.

Instead of developing its own *publish/subscribe* interface, SWES leverages WS-Notification from OASIS to realize the according functionality. This approach is in line with the developments specified by the Sensor Event Service, successor of SAS (Section 3.3.4).

##### Revised Identifier Handling

In the first generation of SWE service specifications, the way that identifiers (of sensors, observed properties, features *etc.*) were used was not harmonized. The SWE Service Model therefore established guidelines for identifier modeling and encoding. In the conceptual model, properties with the purpose of identifying certain objects are modeled as the type of the according object. For example, a *DescribeSensor* request has a *procedure* property of type *OM_Process* which identifies the sensor/process whose description shall be retrieved. From an object oriented programming perspective, this approach is like using object pointers to identify a given object. In the XML implementation of the conceptual model, such object identifying properties are represented as Unified Resource Identifier (URI) so that they are able to store the identifier value. Consequently each resource—such as a sensor, feature or offering—that needs to be identifiable in SWE service models gets a URI. Going a step further, it is recommended to use Unified Resource Locators (URLs), a subtype of URI, to identify such resources. Following this approach, resource identifiers can be easily de-referenced into the actual resource representation. This facilitates the integration of SWE 2.0 concepts into the Linked Open Data Cloud [68] and allows the definition of REST APIs to sensors, features *etc.*, which realizes the idea of a Web of Things (Section 2.4) in a standardized way.

##### Model Mapping

The service specifications of the first generation SWE primarily concentrated on the definition of an XML schema which reflected the service functionality. In the new generation SWE, first a conceptual service model is defined (using UML notation), before an XML implementation of that model is specified. That way, also other forms of implementations of the conceptual model are possible (e.g., a JSON implementation). The SWE Service Model uses a revised version of the GML Application Schema Encoding Rules [59] which enable a full mapping between the conceptual model and its XML implementation. This approach follows the Model Driven Architecture concept. Based on what is defined in the SWE Service Model, both SOS 2.0 and SPS 2.0 make use of the model mapping approach to achieve consistent service models. In fact, as discussed above, also the new specifications SWE Common 2.0 (Section 3.2.1) and O&M 2.0 (Section 3.2.2) define a separate conceptual model and a mapping to its XML implementation.

##### SOAP Binding Introduced

In many IT infrastructures, SOAP [69] enables communication with web services. In its basic form, SOAP simply adds a well-defined envelope around the XML encoded operation request, without any other implications. This resembles the binding style of the well-known OGC standards (e.g., Web Mapping Service, or Web Feature Service) which is based on communication of plain XML operation requests and responses but without any surrounding envelope. If additional communication functionality, such as reliability or security mechanisms, need to come into play, SOAP can be regarded as an enabling and proven technology.

In SWE 1.0, the SOS and SPS standards were silent about a possible SOAP binding. Information items that are needed to fully enable the SOAP binding of SWE services were missing, such as so called action URIs—both for use with SOAP itself, but also with WS-Addressing [70]—as well as a SOAP fault mapping. SWES defines all these information items in the SOAP binding for its operations. Furthermore, it defines how asynchronous communication as well as publish/subscribe functionality shall be achieved in SOAP bindings of SWE standards (such as SOS 2.0 and SPS 2.0). The corresponding technologies are well established IT standards: WS-Addressing and WS-Notification [71], respectively. To not exclude support for IT deployments that use older versions of SOAP, SWES does not prescribe usage of either SOAP 1.1 or SOAP 1.2.

#### 3.3.2. Access to Observations and Sensor Metadata

Standardized access to sensor observations and sensor metadata is provided by the Sensor Observation Service (SOS). The service acts as a mediator between a client and a sensor data archive or a real-time sensor system. The heterogeneous communication protocols and data formats of the associated sensors are hidden by the standardized interface of the SOS. Sensor data requested by a client are returned as observations. The interface of the SOS supports access to heterogeneous sensor types, stationary as well as mobile sensors which gather their data *in-situ* or remotely. Currently, the development of the second version of the SOS specification [72] has finished the public comment phase, and is about to be submitted to the standard approval process. The main advancements from SOS 1.0 to SOS 2.0 are outlined in Table 3 and described in the following.

##### Restructuring of the Specification

By aiming at a clearer structure of the specification document, SOS 2.0 divides its operations and functionalities into a *core* and its *extensions*. The *core* comprises the mandatory operations for retrieval of the service metadata and its content (*GetCapabilities*), for accessing observations (*GetObservation*), and for querying sensor descriptions (*DescribeSensor*). The *transactional extension* contains operations for inserting new sensor descriptions and sensor observations. The *result handling* extension specifies operations for insertion and retrieval of pure observation results without observation metadata to increase performance and scalability. The *enhanced extension* amends the SOS functionality by providing optional operations, for example, to enable the retrieval of observed features. Also SOS 1.0 has separated its operations into four distinct parts, so-called *profiles*. However, this terminology was misleading, since a *profile* of a standard is defined as a subset of the base standards requirements [73], and not as a part of a standard.

##### Increased Interoperability

The flexibility and the generic character of the SOS 1.0 interface have been identified as a factor that may reduce interoperability between different SWE based systems. Multiple SOS 1.0 server and client implementations exist and have been used in various applications [74–77]. However, a single client application capable of retrieving and processing observations from various SOS servers from different vendors without according code adjustments is, to the best knowledge of the authors, currently not available. In SOS 1.0, extensive temporal, spatial and thematic filtering functionality as well as missing profiles for the recommended but very generic response formats have hindered interoperability and the implementation of fully compliant software components. Hence, SOS 2.0 introduces a limited set of mandatory temporal and spatial filters for the operations which allow observation or feature retrieval. Every SOS 2.0 compliant server has to support the temporal filters *during* and *equal*, as well as the *bounding box* spatial filter. Client applications are now able to rely on the support of those basic filters. A further restriction and benefit for interoperability is the restriction of the spatial filter by the newly introduced Spatial Filtering Profile which goes hand in hand with the spatial profile of O&M 2.0 (Section 3.2.2). It defines that the spatial filter is applied to the *sampling geometry* of the observations.

Further, SOS 2.0 defines O&M 2.0 as its mandatory and default response format for sensor data. While SOS 1.0 was unclear about the support of other response formats, SOS 2.0 requires that a formally accepted extension of the standard has to define how the service behaves when responding in another format. For sensor metadata, SOS 2.0 recommends the usage of SensorML (Section 3.2.3). TML, named as a potential response format by SOS 1.0, is not mentioned in the specification anymore.

##### KVP and SOAP Binding Introduced

With the aim of facilitating the usage of the SOS, the version 2.0 of the standard adds a lightweight HTTP GET binding for selected operations. The operation parameters are passed to the service as key-value pairs (KVP) in the URL of the service endpoint. Further, a SOAP binding is introduced by extending what has been defined by the SWES specification (Section 3.3.1). The KVP binding is reduced in complexity, but also in functionality, compared to the XML-based SOAP binding. So, while the KVP binding provides more simple access to the SOS functionality, the SOAP binding enables the integration of the SOS in service oriented enterprise architectures.

##### Capabilities Redesign

A step towards simplifying the standard and streamlining the different SWE services is the introduction of the SWES specification (Section 3.3.1) which is the basis for SOS 2.0. For the *contents* section of the capabilities document, SWES defines abstract types which are reused by SOS 2.0 and SPS 2.0. The contents offered by a service are grouped into so-called *offerings*. In case of the SOS it is an *observation offering*. This concept has already been used by SOS 1.0; however, a redesign of the offering type restricts it now to aggregate only the observations gathered by one instead of multiple sensor systems. Formerly, it has been up to the SOS provider to group observations into offerings. This could have been done by different criteria: spatially, thematically (e.g., per sensor or observed property), or temporally. The simple conceptual change of limiting the offering to one sensor eases the set up and the access of an SOS server, since grouping of observations to offerings is not ambiguous anymore with respect to the sensor that generated the observations in that offering.

An important concept within the SWE framework is the *feature of interest*, the computational representation of a real-world entity modeled with a certain set of properties. This could be for example the feature “Gulf of Mexico”. Also, a sampling point “P_42” within the Gulf of Mexico, where a measurement was taken by a certain (maybe mobile) sensor system, is a feature of interest. Both could have properties such as water depth, salinity or geometry. In SOS 1.0 all features of interest of the observations associated with the SOS server needed to be listed for each observation offering. This is helpful to provide clients a list of features for which observations can be requested. However, this listing of all features has been identified as a problem for mobile sensor systems (e.g., a boat taking measurements on the Gulf of Mexico) which create many sampling features (e.g., sampling points) during operation [78]. Those sampling features could accumulate to huge numbers and could increase the capabilities document up to an unusable state. Hence, the SOS 2.0 does not list sampling features anymore, but instead, *related features* are listed in the capabilities of an SOS 2.0 server. The listing of those related features has the purpose of improving the discovery of observations. The SOS provider decides which related features are meaningfully listed. In the example above, this would be “Gulf of Mexico” and not “P_42”.

##### Advanced Feature Retrieval

Clients still need to be able to retrieve a list of sampling features from the SOS. The knowledge about existing sampling features is for example necessary for the construction of queries for sensor observations. For the retrieval of sampling features a separate operation, called *GetFeatureOfInterest*, has already been defined in SOS 1.0. In SOS 2.0 this operation is extended in its parameterization. Not only a specific feature, or features for a certain spatial filter can be requested, but also features which are observed by specified sensors or which carry certain observed properties.

##### Result Handling Redesign

Allowing the retrieval of the pure observation results for a specified timestamp without the complete set of associated observation metadata has already been supported by SOS 1.0. The purpose of this functionality is to allow clients to repeatedly obtain sensor data without the need to receive responses which largely contain the same data except for a new timestamp and result value. This is in particular useful in scenarios with restricted bandwidth or processing power. The SOS 2.0 specification redesigns and simplifies the *GetResult* operation which is used to retrieve pure observation results. In particular, the response from the SOS server containing the results is defined in a more precise way. An additional operation is introduced (*GetResultTemplate*) which returns an exact description of structure and encoding of the results, by making use of SWE Common 2.0 (Section 3.2.1).

A functionality added by SOS 2.0 is the insertion of pure observation results through new operations (*InsertResultTemplate* and *InsertResult*). This allows inserting sensor results into an SOS without the need to repeatedly transmit the entire set of observation metadata. Similar to the result retrieval functionality, this is useful if the communication bandwidth of the client, in this case the sensor data producer, is limited and the other observation metadata is rather static.

Also, the capabilities model of the SOS 2.0 has been improved to better support the insertion of new data to the SOS. A new section of the capabilities document (called *insertion capabilities* section) now states the observation type, result type, feature types, and encodings supported by the SOS server for insertion of observation data.

##### Retrieval of Metadata about Available Data

In SOS 1.0, questions such as “which sensors generated observations for certain observed properties”, or “for which time frames do observations for a particular feature of interest exist” could not be answered without retrieving the actual observation data by invoking a corresponding *GetObservation* request. The capabilities document, and the contained observation offerings, only provide information on the temporal bounding box of all observations associated with the offering. This is still true for SOS 2.0. In order to support the described use cases, an extension accompanies the SOS 2.0 specification, which defines the *GetDataAvailability* operation [79]. The operation enables clients to discover the temporal relationship between given procedures, observed properties and features of interest. The operation contains parameters that can be used by clients to indicate for which period of time these relationships are to be discovered and also to generalize the information about the temporal relationships. The latter is especially useful to decrease the operation’s response size but also to display this information as it is described in [80]. The operation replaces and amends the functionality of the *GetFeatureOfInterestTime* operation that was defined by SOS 1.0.

#### 3.3.3. Tasking of Sensors

Some sensors or sensor platforms support dynamic configuration at runtime. This can be for example the configuration of the sampling rate or the steering of a movable sensor platform. Tasking of sensors in an interoperable way can be done by using the Sensor Planning Service (SPS). The SPS is a web service interface that allows clients to submit tasks to sensors.

The SPS interface aggregates operations covering the complete process of controlling and planning sensor tasks. This contains checking whether a task is feasible for a sensor by using the *GetFeasibility* operation, the submission of tasks (*Submit*), as well as the status tracking of submitted tasks (*GetStatus*). In order to equip clients with sufficient information to formulate tasking requests, the (self-describing) syntax for describing a task can be requested (*DescribeTasking*). The SPS forwards the submitted tasks to the addressed sensor. However, the subsequently gathered data are not collected by the SPS. Instead, the SPS provides a means for querying where the measured data is accessible (*DescribeResultAccess*). The version 2.0 of the SPS standard brings numerous changes in the request and response models of the client-server communication. We will concentrate on the most prominent advancements from SPS 1.0 [66] to SPS 2.0 [81], as listed in Table 4 and described in the following.

##### Harmonization with SWE Service Model and SWE Common Specification

SPS 2.0 reuses both the SWE Service Model (Section 3.3.1), as well as the SWE Common (Section 3.2.1) specification. All SPS 2.0 operations derive from the abstract request type defined by SWES which provides well-defined extension points for future SPS profiles, such as the SPS EO Profile (see below). In addition, the SWES operations *DescribeSensor* and *UpdateSensorDescription* are implemented in the new SPS specification, the first as a mandatory option and the second as optional. SWE Common data components are now used to describe the tasking parameter syntax and to encode tasking parameters in corresponding requests and supersede the data structures used in version 1.0.

##### Redesign of Task Handling and Status Model

The SPS 2.0 defines a new task model, which clearly differentiates the subtle differences between all possible states a tasking request or task can hold. It captures all statuses from the request reception until eventual completion or failure of a task. The initial model in version 1.0 enumerated the task statuses *unknown*, *in operation*, *finished*, *not yet started*, *cancelled*, and *delayed*, but provided a rather loose definition only and no state change semantics have been described. This has changed with the new model. State machine diagrams illustrate the new concept and exactly define the status of a task at any time. The new model is closely aligned with the new notification concept, as all task state changes or re-entries in already hold states are reported.

In version 1.0, it was necessary to retrieve the current status by issuing *GetStatus* requests. The new operations to reserve a task (*Reserve*) and to confirm a reserved task (*Confirm*) are reflected by the status concept as well. Further, the new *GetTask* operation now allows clients to retrieve complete information about a given task or tasking request. Also, the *GetTask* operation is designed to serve as an extension point for future extensions to SPS 2.0.

The status concept has some consequences on the reporting behavior of SPS service instances. The new SPS 2.0 clearly defines syntax and semantics for the status reports of all possible statuses and their corresponding transitions.

##### New Asynchronous Communication Concept

The asynchronous communication concept has changed fundamentally from version 1.0 to version 2.0. SPS 1.0 used the Web Notification Service (WNS) (Section 3.3.4) to realize asynchronous communication between client and server. To use this feature, clients had to register with a WNS upfront and provided the notification endpoint in the various requests. SPS servers sent all results to this WNS, which acted as a forwarding mechanism using arbitrary communication protocols. SPS 2.0 is not tied to WNS anymore, but supports a *publish/subscribe* model. This model defines a number of event types that can be published to interested consumers. Depending on the subscription model implementation, content based filtering and/or channel based filtering is supported.

##### SOAP Binding Introduced

The SPS 1.0 model only specified encodings appropriate for use of HTTP GET transfer of operation requests (using key-value pairs (KVP) passed to the service in the URL), and for use of HTTP POST transfer of operations requests (using plain XML or KVP encoding). SPS 2.0 introduces a SOAP binding based on what has been defined in the SWES specification (Section 3.3.1). Additional bindings can be added through extensions to the baseline specification.

##### SPS EO Profile

The fundamental changes to the SPS specifications have been reflected in a new version of the earth observation profile for SPS [82]. This profile is still *sui generis*, though it is expected that new profiles will be developed in the future focusing on resource-oriented implementations of the SPS model.

#### 3.3.4. Eventing and Alerting

This section covers the specifications which have been developed in context of the SWE initiative to realize push-based and asynchronous communication. This enables *eventing*, *i.e.*, the automatic publication of data that is of interest for the user (without him having to repeatedly pull for that data), and *alerting*, which incorporates the publication of more significant data to the user who is supposed to react in a domain or application specific way upon receipt of this kind of data. In contrast to classic SWE services, e.g., the SOS (Section 3.3.2), which use the request-response communication pattern, the services described in this section are based on the *publish/subscribe* pattern. This allows disseminating data (events) as soon as possible and without the need to periodically request them. Especially *in situ*ations when the update rate of the data source is unknown, such push-based systems can be of high value. The basic functionality of such services is allowing consumers to subscribe for notifications and the ability to send proper notifications to subscribed consumers [83]. Enhanced services may also be able to act as a notification broker and thus be notification consumers themselves.

The Sensor Alert Service (SAS) [65] and Web Notification Service (WNS) [67] were the first services in SWE 1.0 to enable alerting. This functionality was revised and enhanced during the development of the SWE 2.0 standards. The table below and the following sections describe the advancements and changes which have been achieved within the development to the new SWE framework.

##### Sensor Alert Service

The OGC Sensor Alert Service (SAS) was the first specification developed at the OGC to handle a push-based access to sensor data. However, its specification [65] did not reach the status of an approved OGC standard. The SAS allows consumers to subscribe to sensor data with some filter criteria such as a bounding box or a simple measurement value threshold. Notifications from and to the SAS are sent via XMPP [84] and encoded in a simple format defined in the SAS specification. The SAS was aligned to some extent to the output of SensorML 1.0 described processes. Alignment with O&M 1.0 was unfortunately not realized. Furthermore, the SAS specified its own *publish/subscribe* operations rather than reusing existing IT standards (e.g., [71,85]) that provide the required functionality, thereby not facilitating interoperability. The development effort of the community was thus redirected to completely revise and improve the SAS, which led to the Sensor Event Service specification.

##### Sensor Event Service

The Sensor Event Service (SES) [86] is an OGC discussion paper and an experimental successor of the SAS. These two specifications differ in several points. In general, the idea of the SES development has been to strengthen the use of existing standards and specifications and leverage them instead of defining service specific solutions. One of these changes is the use of the OASIS WS-Notification (WS-N) standard for the definition of the service operations needed for a publish/subscribe communication. This suite of standards defines operations for subscription handling and notifications (WS-BaseNotification) [71], for the brokering of notifications (WS-BrokeredNotification) [87] and for the use of event channels (WS-Topics) [88]. These event channels allow grouping of notifications with respect to a specific topic, for instance weather forecasts. Instead of defining the filter for forecasts in each consumer’s subscription, a consumer can simply subscribe for all notifications on the weather channel.

The SAS specific encoding of sensor measurements has been replaced by using the O&M standard (Section 3.2.2). This allows providing additional metadata with each measurement and thus enhances the interoperability between the different SWE services. Especially integrating a Sensor Observation Service and a Sensor Event Service in the same system is much easier due to the use of the same data encoding.

The language for the subscription filter definition has also been updated. The SAS used its own integrated filter language which only offered limited functionality. As a basic filter language, the SES requires the support of XPath [89] to perform filtering based on XML patterns within the notification. In addition, two optional filter languages are supported by the SES: the OGC Filter Encoding (FES) [90] and the Event Pattern Markup Language (EML) [57]. The former is also used in the Web Feature Service (WFS) and is more expressive than the filters offered by the SAS. The EML builds upon the FES. It enables (Complex) Event Processing (CEP) [91] functionality such as the detection of relations between events, the use of data windows to include multiple notifications in an event pattern and the possibility to even derive new information in contrast to pure filtering.

##### Event Pattern Markup Language

The SAS filters incoming sensor data one by one. A piece of data either matched the filter criteria or it did not. Correlation of multiple sensor measurements is not possible, preventing detection of interesting patterns as well as derivation of higher-level information from the measurement stream. The Event Pattern Markup Language (EML) [57,92] was developed to support this event processing functionality. As outlined before, the Sensor Event Service was the first prototype within OGC that supported EML. The language supports fundamental event processing features, such as views upon event streams, select functions, guard conditions as well as simple and also complex event pattern constructs that for example allow investigation of event causality.

##### Web Notification Service

The OGC Web Notification Service (WNS) [67] is a specification which was developed in parallel to the SAS. The WNS acts as a protocol transducer for a ‘last mile mode’ of notifications. It is able to receive notifications and to forward them to registered clients via different protocols such as email or SMS. This way one can ensure that important notifications reach their destination as soon as possible. The WNS has also been used in combination with the SPS 1.0, to inform the client about the progress of a task that the SPS performs. This strong dependency on WNS is no longer the case for SPS 2.0 (Section 3.3.3).

#### 3.3.5. Discovery

The SWE framework supports the flexible integration of all kinds of sensor data sources into applications. However, the availability of interoperable sensor data sources must be complemented by discovery solutions that allow users to find the data they need for solving their questions and tasks [93]. In conventional SDIs consisting of servers providing maps, coverages or geometric features, this is usually covered by the OGC Catalogue [94]. Due to the specifics of sensor networks, this approach is not directly applicable for the Sensor Web [95]. On the one hand, there are different metadata models: SensorML within the Sensor Web and for example ebRIM [96] used by catalogues. On the other hand, the often very dynamic structure of sensor networks creates further challenges that are not reflected by the OGC Catalogue interface. Further questions comprise the automatic collection of sensor metadata and semantic problems such as the description of the phenomena a sensor is observing. In order to address these challenges, the approaches described in Table 6 have been developed.

The enhancements of the SWE framework in order to enable sensor discovery address both the information model (Section 3.2) as well as the interface model (Section 3.3). Within the SWE information model the discovery enhancements mainly address the provision of sufficient SensorML based sensor metadata and the mapping of those metadata elements to catalogue information models such as ebRIM. For extending the interface model two kinds of web services are discussed. The Sensor Instance Registry (SIR) for harvesting, managing and transforming sensor metadata as well as the Sensor Observable Registry (SOR) for managing the semantics of the phenomena observed by sensors.

##### SensorML Profile for Discovery

The SensorML standard (Section 3.2.3) is the recommended encoding for sensor metadata within SWE. Due to the very broad range of use cases and sensor types that are supported by the SWE specifications, SensorML has been designed in a very flexible way. Thus, SensorML can be used for describing sensors ranging from fixed stationary devices such as weather stations to complex data acquisition systems for aerial images. For enabling sensor discovery this flexibility of SensorML induces certain challenges: SensorML defines only very few mandatory elements so that it is not ensured that all metadata elements necessary for enabling sensor discovery are available. Furthermore, SensorML allows different ways for encoding the same metadata items. Consequently it becomes difficult to automatically process SensorML documents and to index them in a sensor catalogue. As a solution, the SensorML Profile for Discovery has been developed [97]. Based on a number of formalized rules (expressed in Schematron [98]), this profile defines a minimum set of metadata elements and their structure that need to be provided if a sensor shall become discoverable.

##### SensorML-ebRIM-Mapping

While SensorML is used in SWE to encode sensor metadata, catalogues used in conventional SDIs are based on different models (e.g., the ISO 191xx standards or ebRIM [96]). Hence, existing OGC Catalogues are not directly able to handle SensorML encoded metadata. Instead, it is necessary to map SensorML based sensor metadata to the existing Catalogue information models so that the discovery of sensors using the OGC Catalogue becomes possible. An according mapping between SensorML and the ebRIM model is described in [99]. Based on the SensorML Profile for Discovery [97], this mapping describes how the different discovery relevant elements of SensorML can be put into an ebRIM model. Practically, this mapping allows deriving an XSLT [100] transformation that can be applied when inserting SensorML based metadata into OGC Catalogue instances.

##### Sensor Instance Registry

The SIR interface [101] provides functionality to collect, manage, transform and transfer sensor metadata. It is intended to close the gap between the SensorML based SWE world and conventional SDIs. In order to achieve this aim, the SIR provides functionality to:

- collect sensor metadata (*i.e.*, automatic harvesting of SensorML documents and manual insertion of sensor metadata)
- provide extended discovery functionality based on the metadata provided through SensorML
- manage status information of sensors (e.g., finding all sensors with a critical battery level or automatic notification if a sensor reaches a critical state)
- transform SensorML-based sensor metadata into conventional Catalogue information models and push the transformed metadata sets into OGC Catalogue instances

In the future it is expected that the functionality of the SIR interface will partly be covered by other existing SWE services (e.g., SOS for retrieving sensor status information and the SES for filtering sensor status updates).

##### Sensor Observable Registry

When searching for sensors a very important parameter is the phenomenon observed by a sensor. Usually such parameters are expressed within SensorML documents through some kind of identifier (*i.e.*, URIs). In order to support users when dealing with identifiers pointing to phenomenon definitions, the SOR interface has been designed [102,103]. It provides functionality to:

- retrieve a list of known phenomenon identifiers so that a user can select those identifiers that fit to his needs
- resolve phenomenon identifiers (*i.e.*, returning a dictionary entry describing what a certain phenomenon identifier means)
- find related phenomena so that sensor discovery requests can be semantically enhanced (e.g., searching for all sensors that measure some kind of temperature)

In the future it is expected that more generic approaches will be developed that support not only the handling of phenomenon definitions but also other kinds of names and identifiers (e.g., for sensor types, units of measurement, *etc.*).

## 4. Applying SWE

The previous chapter described the different SWE specifications and discussed the changes made in the new generation SWE. This chapter first describes an example use case of a Sensor Web infrastructure. Next, selected research projects and applications are presented which have utilized, evaluated and enhanced the SWE framework in the past years.

### 4.1. Example SWE Deployment

**Figure 5.**
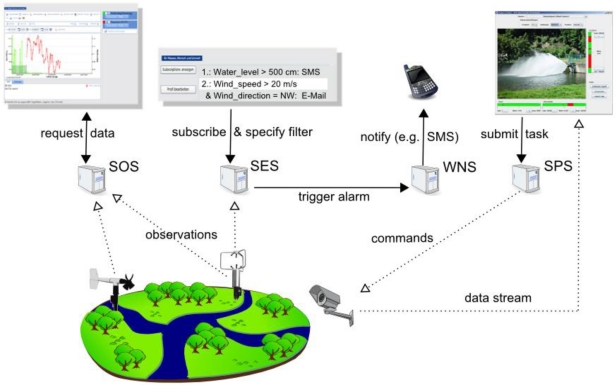
Deployment scenario for SWE services.

An exemplary case-study of the application of SWE in a real-world hydrological deployment scenario is shown in Figure 5 and is based on what has been described in [104]. The SWE services are applied to manage a network of hydrological sensors (e.g., water gauges, weather stations, or cameras observing critical facilities) by providing access to sensor data (Section 3.3.2), by realizing event handling (Section 3.3.4), and by enabling interoperable tasking of sensors (Section 3.3.3). The described deployment can be easily adapted to other real-world deployments of sensor networks.

Observations from the various sensor resources out in the field are inserted to an SOS. The figure shows a direct connection between sensor and service. However, in real world applications the raw data measured by sensors is first processed, enriched and encoded as O&M before it can be inserted to the SOS. Hence, in real world deployments there are usually data acquisition systems and middleware components located between sensors and SWE services. Once the observations are uploaded to the SOS, applications can retrieve the data through the standardized interface and can visualize it for example as time series charts or on maps.

If a client is only interested in particular data which matches some defined filter criteria, e.g., the exceedance of a threshold, it can subscribe to an SES. The sensor data is continuously published to the SES and in case a specified filter criterion is matched, the SES forwards the data to the client. Clients can also register for alarms if certain events occur. In that case, the SES triggers a WNS to notify the client via a defined communication protocol. For example, a user can receive a notification via SMS or email if the water level at a gauge station is above 5 meters.

Finally, the SPS is utilized to task sensors. For example, the SPS can be used to task cameras at certain points of interest along a river course (e.g., a dam or a water gauge). The cameras can be rotated or zoomed and the real time video stream can be accessed by the client through another means of data access.

### 4.2. SWE Projects and Applications

In recent years, SWE based Sensor Web infrastructures have been deployed in various projects and applications which demonstrated the practicability and suitability of the SWE standards. In the following, we present a non-exhaustive selection of such SWE projects and applications. Table 7 gives an overview and further information about the selected projects, for example about which SWE service types have been used.

The architecture of the OSIRIS project (*http://www.osiris-fp6.eu/*) has been based on SWE standards [75]. SOS, SAS and SPS were enhanced and used in a broad range of use cases ranging from forest fire fighting [105], to air pollution monitoring by attaching sensors to busses [106]. The SANY project (*http://sany-ip.eu/*) [107] dealt with environmental and risk management. It aimed at improving the interoperability of in-situ sensors and sensor networks to enable the reuse of data and services [77]. Within the GENESIS project (*http://www.genesis-fp7.eu/*), the SWE services have been applied in practical scenarios ranging from air quality to fresh and coastal water quality. A special focus has been put on sensor discovery and concepts for event based architectures. The INTAMAP project (*http://www.intamap.org/*) developed a real-time processing service for the interpolation of observations provided via SOS [108]. The EO2Heaven project (*http://www.eo2heaven.org/*) contributes to a better understanding of the complex relationships between environmental changes and their impact on human health by building a spatial information infrastructure which applies SWE services to monitor human exposure to environmental pollution and for an early detection of infections. The ESS project (*http://www.ess-project.eu/*) develops an infrastructure based on SOS, SPS, and SES to provide real-time information to crisis managers during abnormal events to improve the management between forces on the ground (e.g., police and firefighters) and the control centers. The UncertWeb project (*http://www.uncertweb.org/*) is dedicated to integrate quantified uncertainty into web based environmental model chains which also involves the incorporation of the uncertainty assessment into O&M and SensorML.

CSIRO’s South Esk Hydrological Sensor Web deals with monitoring the water cycle in Tasmania and in particular forecasting the short-term river flow. Within the test bed, SWE standards have been used to develop new hydrological and water resource management tools [109].

The Advanced Fire Information System (AFIS) project [110] combines in-situ measured sensor data (e.g., from weather stations) and remote sensing data to detect wild fires in South Africa. As soon as the power supply infrastructure (power lines or pylons) is endangered, an automatic notification of responsible persons is triggered so that damage to transformers can be prevented.

The OOSTethys project (*http://www.oostethys.org/*) has developed software components to leverage oceanographic research. The aim has been to integrate heterogeneous ocean observing systems such as the application oriented *Integrated Ocean Observing System* (IOOS) and the research-oriented *Ocean Observatories Initiative* (OOI) by utilizing SWE and other standards [111]. OOSTethys lead two test-beds under the umbrella of the OGC, the Oceans Science Interoperability Experiment (Oceans IE) 1 & 2, to evaluate and advance the SOS. The two test-beds produced reference implementations as well as engineering reports and best practices [112,113] about how to implement and utilize SOS services. Similar to the Oceans IE 1 & 2 test-beds, but based on other thematic domains, the Groundwater IE (*http://www.opengeospatial.org/projects/initiatives/gwie*) as well as the Surface Water IE (*http://www.opengeospatial.org/projects/initiatives/swie*) have been conducted.

Within the GITEWS project (*http://www.gitews.de/)* [114], a tsunami early warning system for the Indian Ocean has been developed based on SWE. The system integrates terrestrial observation networks of seismology and geodesy with marine measuring sensors, satellite technologies and pre-calculated simulation scenarios.

The SoKNOS project (*http://www.soknos.de/*) developed concepts to support governmental agencies, private companies, and other organizations in handling disastrous events. The SOS was used to integrate live sensor data into the situation map of a disaster management organization [76]. Additionally, a concept for tasking mobile sensors and optimizing their coverage based on interpolation errors was developed using the SPS [115].

The h2.0 project [116] has aimed at developing community-driven services for monitoring the water supply in East Africa. This is achieved by developing a Human Sensor Web in which user generated content submitted via cell phones is integrated with SWE services to inform communities about water supply. Also a user-driven platform is developed within the GeoCENS project (*http://www.geocens.ca/*). The SWE based platform shall serve for biogeoscience researchers to store and share ground-based sensor array data regardless of their location on a scale not currently possible.

A small selection of applications using the SWE framework includes a GIS expert system which can be utilized for near-real-time hazard monitoring [117], or the integration of sensor information into pervasive advertisement (e.g., digital signage, mobile phones) to show live data about the environment (e.g., weather forecast) and adapt information presentation to the context, as determined by sensors (e.g., advertisement for ice cream when the sun is shining) [118]. In Taiwan, debris flow caused by the torrential rainfall is a severe problem. An information system for debris flow monitoring stations has been built on SWE standards in combination with grid computing technologies [74].

## 5. Challenges and Future Work for SWE

In the following we describe open challenges and future work in the area of Sensor Web Enablement. These challenges relate to seven topics: the improvement of interoperability, the facilitation of sensor and service integration, the advancement of Sensor Web eventing concepts, the assessing of observation metadata such as uncertainty, the realization of a Human Sensor Web and the integration with online social networks, as well as the enablement of the Semantic Sensor Web. The list of stated challenges does not claim to be exhaustive, but is supposed to help understanding some open problems in this research field.

### 5.1. Increasing Interoperability

While SWE has proven its applicability in a wide variety of projects and applications, it is still not yet widely used in productive systems. One reason for that is the generic nature of the standards which is required to be applicable for a broad range of domains. The SOS (Section 3.3.2), as an example, is intentionally defined for all kinds of sensor resources, ranging from thermometers to satellites. The required flexibility allows on the one hand the integration of heterogeneous sensors, but on the other hand it leaves certain elements generic to fulfil the flexibility requirement. An example is the type of the observation result which is not restricted to a specific type in the basic O&M model (Section 3.2.2). This makes it difficult to implement generic and interoperable clients capable of dealing with different SOS implementations as the result type is not known *a priori* and not restricted to a certain subset. A useful approach to tackle this problem is to define domain specific profiles. The Water Markup Language 2.0 (WaterML 2.0) [119] is such a profile for the hydrological domain. It restricts the result of an observation to a time series type and defines several other restrictions on sensor types, phenomenon types and allowed types for the feature of interest. In future, further profiles need to be defined to enhance interoperability within domains.

Though the SWE standards can be used in complex scenarios, most of the currently available deployments are providing data from fixed *in-situ* sensors. For those simple kinds of sensors, a lot of the above described flexibility in the standards is not needed. Thus, a *lightweight SWE profile* for stationary *in-situ* sensors would ease the implementation and would enhance interoperability.

### 5.2. Facilitating the Integration of Sensors and Services

The ability to dynamically integrate sensors is still an unresolved challenge within SWE. An on-the-fly integration of sensors into the Sensor Web with a minimum of human intervention is not straight-forward with the given methods. Especially in hazard or disaster situations, a live deployment or densification of sensor networks and an *ad-hoc* integration of those sensors into the Sensor Web to allow multiple parties an easy access and usage of the sensors must be enabled.

The SWE services are intentionally designed from an application-oriented perspective. Currently, sensors are usually connected by manually building adapters for each pair of web service and sensor type. Those adaption efforts are a key cost factor in large-scale sensor network systems [120]. Bridging this *interoperability gap* [121] between the Sensor Web layer and the lower-level sensor layer can be addressed from two directions.

First, the interoperability on the sensor layer can be improved which is addressed by several standardization efforts. An example is the IEEE 1451 family of standards [122], a universal approach to connect sensors to diverse networks and systems. Another example is the PUCK protocol that extends the sensor firmware, and provides a means to retrieve a universally unique identifier, metadata and other information from the device itself through its communication interface [123]. It is envisaged to bring PUCK into the OGC standardization process.

However, in today’s real world applications a huge variety of sensor protocols (standardized or proprietary) are utilized. Thus, several projects are addressing the interoperability gap from the opposite direction, by introducing mechanisms to abstract from the variety of sensor protocols (e.g., AnySen [107], Sensor Abstraction Layer [124], or Sensor Bus [29]). However, those approaches still need manual creation of sensor adapters.

A promising, universal approach is the Sensor Interface Descriptor (SID) model [62] which extends SensorML (Section 3.2.3). It can be utilized to formally describe a sensor’s protocol. Graphical editors are in development [125] which can be utilized to create instances of the SID model. The generated sensor interface description is used as a platform independent sensor driver which contains the necessary information to integrate a sensor on demand by translating between sensor protocol and Sensor Web protocols. A still remaining open challenge is to include such a universal approach for the sensor interface abstraction, e.g., SID, in the standardization process and to develop tools which facilitate its usage. Semantic challenges which have to be tackled to enable an automatic sensor plug & play are discussed in [126]. Difficulties lie in establishing the semantic matching between SWE concepts used for modelling sensors and observations (e.g., feature of interest or observed property) and the constructs of the lower sensor network layer.

### 5.3. Extending Sensor Web Eventing Concepts to a Common Event Architecture

The new generation SWE significantly improves the specifications on alerting and event notification of SWE 1.0 by augmenting the event filtering functionality. Thereby, the work focused on the needs of the Sensor Web. Recent developments indicate that the need for a common event architecture, not only for the Sensor Web, but for SDIs in general, is growing. First concepts for realizing publish/subscribe functionality and languages for enabling event processing within such a common event architecture have been developed [127,128]. The SES and EML (Section 3.3.4) already represent first steps towards this common functionality. Also, SWES (Section 3.3.1) and SPS (Section 3.3.3) reuse common publish/subscribe interfaces defined by WS-Notification [71]. Those standards only define the events and event channels that belong to the respective Sensor Web sub domains.

It is envisaged that future SWE standards will reuse the concepts and functionalities provided by a common event architecture. Then, extensions and profiles need to be created that capture the eventing aspects that are specific to the Sensor Web domain. That encompasses sensor event type and event channel definitions as well as functionality specific to processing sensor events.

### 5.4. Assessing Data Quality, Provenance and Uncertainty

Knowledge about the quality, provenance and uncertainty of sensor outputs is essential for making the right decisions based upon observations. At the moment, such information is often missing in observations and there is no unique way of how to incorporate it. As observations are usually inputs to environmental models, one aspect is to define a common method for integrating uncertainty into observations encoded as O&M. The Uncertainty Markup Language (UncertML) [129] is one approach which can be used as a basis for the integration with O&M. This would ensure that uncertainty information is communicated in a common way within Sensor Webs.

### 5.5. Realizing the Human Sensor Web and Integrating Social Networks with the Sensor Web

Since 2004, new kinds of Web applications have been created. They are called Web 2.0 applications, because they are fundamentally different from the previous generation of Web applications (*i.e.*, Web 1.0 ones). Web 2.0 applications build online social networks to inter-connect users, treat users as information “*prosumers*” (*pro*vider- con*sumer*), enable them to create “user-generated content”, and harness their collective intelligence for innovative applications [130]. Web 2.0 has revolutionized the way today’s users interact with each other and share information on the Web.

By looking at Web 2.0 applications which allow sharing of *geographic* information, Goodchild [131] coined the term Volunteered Geographic Information (VGI) and proposed to extend the notion of sensor networks by incorporating humans as sensors. Examples of such applications enable users to share information about their bird sightings (see *http://www.birdpost.com*) or allow uploading measured weather data to an online social network [132]. Other applications allow their users to contribute to earthquake science by either filling out a Web form about the intensities of shaking and damage caused by an earthquake [133] or by contributing the built-in accelerometer of one’s computer to a national seismic sensor network [134]. Based on observations of flood events from the affected population, Poser *et al.* [135] develop methods for the quality assessment of such human generated observations for rapid loss estimation.

Thereby, it can be distinguished between *human sensed observations* (such as textual descriptions) and *human collected observations* gathered by sensors which are carried by a human (e.g., measurements performed by smart phones). The aim of the *Human Sensor Web* [136] is to integrate those two kinds of human observations by utilizing the SWE framework of standards. An example for a Human Sensor Web application which incorporates SWE is a water availability monitoring system for improving the water supply in East Africa [116]. Challenges with regard to the Human Sensor Web are broad, ranging from the design of ergonomic user interfaces, stimulating incentives of people to participate in the Human Sensor Web, handling of human cognition and resulting uncertainties, ensuring security, privacy, and trust, as well as dealing with the unstructured information provided by human observers.

Analyzing and utilizing the social connections between users of online social networking platforms to enhance the Sensor Web is another emerging research direction. New models and architectures need to be designed in order to build a social networking-based Sensor Web. New algorithms need to be developed in order to exploit the social networks’ underlying social graphs to enhance the Sensor Web. For example, Liang [137] discussed the long tail phenomenon of the Sensor Web, and developed GeoCENS [138], a SWE-based online social network allowing scientists to share their sensor data. Based on the GeoCENS social network graph, a geospatial folksonomy and a collaborative tagging system have been developed [139] that recommend sensors and datasets according to a user’s geographical area of interest.

### 5.6. Enabling the Semantic Sensor Web and Linked Sensor Data

Research on the Semantic Sensor Web [140] investigates the role of semantic annotation, ontologies, and reasoning to improve Sensor Web functionality such as sensor discovery and sensor integration. It combines OGC's vision of a Web of sensors with the reasoning capabilities of the Semantic Web [141]. Related work in this field includes methods for linking geosensor databases with ontologies [142], a semantically-enabled Sensor Observation Service (SemSOS) [143], an analysis of the challenges to realize semantic sensor plug and play [126], or the semantic annotation of sensor services with terms from ontologies [144]. Recent approaches to enrich geospatial services with semantics include an OWL-Profile for the Catalogue Service Web (CSW) suggested by Stock *et al.* [145] and the development of a transparent semantic enablement for SDIs [146]. The latter approach defines specific profiles for Web Processing Service (WPS) and CSW to serve functionality for reasoning and ontology look-up, respectively.

Ontologies need to serve as the basis for semantic reasoning. Hence, thoroughly defining models for sensors from an ontological perspective is a challenging research task. Various research groups started to specify sensor, stimuli, and observation ontologies. Examples include the Semantic Web for Earth and Environmental Terminology (SWEET) (*http://sweet.jpl.nasa.gov/ontology*) focusing on modelling of observed properties, observation based ontologies influenced by O&M [147,148], and a sensor-centric ontology with a strong relation to SensorML [149]. Also, there are domain-specific ontologies, such as the approach of the Marine Metadata Interoperability project (*http://marinemetadata.org/*), which is particularly designed for oceanographic sensors [150], but could be adapted to other domains in the future. Promising is the observation-centric ontology recently developed in a consensus process within the W3C Semantic Sensor Network Incubator Group (*www.w3.org/2005/Incubator/ssn*) [151].

Another important research direction in the context of enabling the Semantic Sensor Web is applying the Linked Data principles to make sensor resources available on the Linked Open Data Cloud [68]. Those sensor resources are identified by URIs which can be dereferenced over simple HTTP calls to retrieve representations of those resources in machine-interpretable formats such as RDF [152]. Research on linked sensor data has for example addressed the design of meaningful URI schemes for sensor resources [153] as well as the filtering and retrieval of linked sensor data through RESTful interfaces [154].

The presented approaches of the Semantic Sensor Web and linked sensor data build a solid basis for future efforts in this research area. Much work still remains to be done. From the perspective of SWE, a central challenge is semantic enablement of SWE specifications and incorporation into the OGC standardization process. This is needed to pave the way to utilization of those methods in real world applications.

## 6. Conclusions

This work comprehensively describes the new generation of OGC’s Sensor Web Enablement (SWE) framework of specifications by particularly focusing on the performed changes compared to the first generation of SWE. The gained experiences from applying and deploying SWE in several projects during the last years were used to develop this new generation of SWE. This evolvement of SWE has taken into account limitations, difficulties and suggestions for enhancement that arose from its practical application.

Within SWE’s information model, several significant changes were made. The SWE Common data model has been extracted from the SensorML specification and is now defined in its own standard document, which emphasizes its gained importance. On the other hand, the development of TML can be considered as discontinued. The model design of the second version of the O&M specification has been brought into the ISO standardization process to strengthen its reliability and relevance. Profiles for certain aspects of the information model are emerging. An example is WaterML 2.0, a hydrology profile for O&M, as well as the SensorML profile for discovery.

An important addition to the SWE architecture is the introduction of the SWE Service Model specification which defines a common model consisting of basic types for requests and responses as well as the Capabilities document of SWE services. Also, well-established IT standards such as SOAP and WS-Notification have been incorporated. SOS 2.0 and SPS 2.0 are now based on this common model which reduces redundancy and shall facilitate the implementation of those standards. Further, SOS and SPS have been evolutionary updated. The specifications are now modularized and consist of a core, extensions and profiles. Significant enhancements have been made in the field of eventing and alerting. Whereas filtering and alerting functionality was previously covered by the SAS specification, the new concepts of SES and EML promise a more powerful solution. While the event filtering functionalities of the SAS were rather limited, SES and EML allow the usage of techniques such as Complex Event Processing and Event Stream Processing to achieve a new level of filter capabilities including the consideration of time, the logical conjunction of filter rules and more advanced geographical filtering. Finally, the SWE framework is completed by first approaches to integrate SWE and Catalogues in order to make sensing resources discoverable.

Despite these advancements, there are still research challenges in the field of Sensor Web Enablement to be tackled in future, as identified in this article. An important topic is to increase the interoperability of SWE components and to facilitate the utilization of the specifications by developing profiles that reflect the requirements of certain user groups or thematic domains. Further, the gap between low level sensor interfaces and the interfaces of Sensor Web services needs to be closed, in order to integrate sensors more easily into Sensor Web infrastructures. Of a broader scope than the SWE framework are the eventing concepts defined by the SES and EML specifications. In future, they will be extended to an OGC wide event architecture. Also, the application of SWE to build a Human Sensor Web and the integration of Sensor Webs with online social networks are challenges needed to be addressed. Finally, the ongoing work on the enabling of a Semantic Sensor Web and applying the Linked Data principle to sensors and sensor data are promising and involve interesting research challenges.

In summary, the new generation of SWE specifications provides a significant step forward. As the development of the new specifications has been strongly driven by the experiences gained from applying the first generation of SWE, it is expected that the acceptance of the SWE architecture in practice and the number of SWE applications will increase further. The new generation of SWE can be considered as an evolutionary advancement of the existing standards baseline. Thus, users of the first generation of SWE specifications will easily be able to upgrade their existing systems to the new generation of SWE.

## Figures and Tables

**Table 1.** Major changes from the first to the new generation of the SWE information model.

**Specification**	**Description of change**
SWE Common 2.0	Extracted to separate specification document Separation of conceptual model and its implementation Independence from Geography Markup Language Improved definition of data stream encodings Extension point mechanism
O&M 2.0	Separation of conceptual model and its implementation Conceptual model has become ISO standard Spatial profile has been added New observation properties (e.g., *valid time* and *related observation*) Revision of terminology (e.g., *sampling time* renamed to *phenomenon time*) Dropping of *observation collection* type
SensorML 2.0 (*in progress*)	Property inheritance mechanism (*under discussion*) Sensor interface description (*under discussion*) Profiles have been defined (e.g., SensorML for Discovery)
TML	*No evolvement*. Not mentioned in the specifications of the new generation
EML	New specification which adds functionality for complex event processing

**Table 2.** Major changes introduced by SWE Service Model 2.0.

**Specification**	**Description of change**
SWE Service Model 2.0	Common capabilities content model: Property inheritance mechanism to decrease size of capabilities documents Introduction of *abstract offering* as base for offering types of specialized services Extensibility points for operation requests, responses and other data types Improved sensor description management: a sensor description is format agnostic according to O&M design principles and facilitates revision management definition of a common *DescribeSensor* operation for retrieval of sensor descriptions definition of a common *UpdateSensorDescription* operation for modification of existing sensor descriptions Conceptual models for sensor insertion and deletion operations (*RegisterSensor* and *DeleteSensor*) Basis for SWE service eventing introduced by defining a notification package to support publication of SWE service events and provision of notification metadata Revised identifier handling to harmonize identifier usage across the SWE specifications Definition of rules that enable automatic mapping between conceptual model and XML Schema implementation SOAP binding introduced

**Table 3.** Major changes of SOS 2.0.

**Specification**	**Description of change**
SOS 2.0	Restructuring of the specification by separating into core and extensions KVP binding introduced Increased interoperability: Mandatory set of operators and operands for temporal and spatial filters Spatial Filtering Profile defines interoperable access to spatial observations O&M as default and mandatory response format for observations Capabilities redesign: One sensor per observation offering Related features instead of all features of interest are listed in Capabilities Result handling redesign: New operations for result insertion (*InsertResult* and *InsertResultTemplate*) New operations for result retrieval (*GetResult* and *GetResultTemplate*) Advanced feature retrieval by extending the *GetFeatureOfInterest* operation Removed operations for the retrieval of types (*DescribeObservationType*, *DescribeResultModel*, and *DescribeFeatureType*)
SOS 2.0—Get Data Availability Extension	Added extension for the retrieval of metadata about available data (*GetDataAvailability* operation)

**Table 4.** Major changes of SPS 2.0.

**Specification**	**Description of change**

SPS 2.0	Harmonization with SWE Service Model and SWE Common specification: Implementation of operations according to SWE Service Model Tasking parameters and tasking parameter descriptions based on SWE Common
	Redesign of task handling and status model: Clear definition of state change semantics New operations: *GetTask*, *Confirm*, *Reserve* Advanced status reporting New asynchronous communication concept SOAP binding introduced

SPS EO Profile	SPS Earth Observation Profile Update

**Table 5.** Major changes of Eventing and Alerting architecture.

**Specification**	**Description of change**
SES (as the successor of SAS)	Integration and leveraging of existing standards for realizing publish/subscribe interface and encoding event data (e.g., WS-Notification and O&M) Enhanced filtering and processing functionality
EML	Enables Event Processing functionality for detecting patterns in (sensor) data streams and deriving new, higher-level information.
WNS	*no changes yet*; may be updated in future

**Table 6.** Major advancements introduced by discovery architecture.

**Specification**	**Description of change**
SensorML Profile for Discovery	Profile of SensorML ensuring the presence of a minimum set of metadata that is necessary for allowing sensor discovery.
SensorML-ebRIM Mapping	Mapping of SensorML elements into the ebRIM Catalogue information model in order to enable the management of sensor metadata by OGC Catalogues.
SIR	Web service interface for managing sensor metadata; this includes the collection of sensor metadata, management of sensor status information as well as functionality for pushing sensor metadata into OGC Catalogues.
SOR	Web service interface for accessing phenomenon definitions and for exploring semantic relationships between different phenomena.

**Table 7.** Overview of selected projects and their utilization of certain SWE services.

**Project Name**	**Funding Source**	**Time Frame**	**SOS**	**SPS**	**SAS**	**SES**	**WNS**	**SIR**	**SOR**

AFIS	ESKOM, CSIR		+		+		+		
South Esk Hydrological Sensor Web	CSRIRO WfHC		+						
GITEWS	BMBF	2005–2008	+						
OSIRIS	EC FP-6	2006–2009	+	+	+	+	+	+	+
SANY	EC FP-6	2006–2009	+	+	+				
Intamap	EC FP-6	2006–2009	+						
OOSTethys	NOAA, NSF	2006–2009	+						
Oceans IE 1 & 2	*unfunded*	2006–2009	+						
SoKNOS	BMBF	2007–2010	+	+					
h2.0	Google.org	2009–2010	+			+	+		
Groundwater IE	*unfunded*	2010	+						
Surface Water IE	*unfunded*	2010–2011	+						
GeoCENS	CANARIE	2009–2011	+	+					
GENESIS	EU FP-7	2009–2012	+			+	+	+	+
ESS	EC FP-7	2009–2013	+	+		+			
EO2Heaven	EC FP-7	2010–2013	+						
UncertWeb	EC FP-7	2010–2013	+						
